# An Exploration of Underrepresentation of Aboriginal Cancer Patients Attending a Regional Radiotherapy Service in Western Australia

**DOI:** 10.3390/ijerph15020337

**Published:** 2018-02-14

**Authors:** Marilyn Lyford, Margaret M. Haigh, Siddhartha Baxi, Shelley Cheetham, Shaouli Shahid, Sandra C. Thompson

**Affiliations:** 1 Western Australian Centre for Rural Health, The University of Western Australia, 167 Fitzgerald Street, Geraldton, Western Australia 6530, Australia; marilyn.lyford@uwa.edu.au (M.L.); margaret.haigh@uwa.edu.au (M.M.H.); Siddhartha.Baxi@genesiscancercare.com.au (S.B.); shelley.cheetham@uwa.edu.au (S.C.); s.shahid@curtin.edu.au (S.S.); 2 Radiation Oncology, South West Radiation Oncology Service, South West Health Campus, Corner of Bussell Hwy & Robertson Drive, Bunbury, Western Australia 6230, Australia; 3 School of Nursing, Midwifery and Paramedicine, Curtin University, Kent Street, Perth, Western Australia 6102, Australia; 4 Centre for Aboriginal Studies, Curtin University, Kent Street, Perth, Western Australia 6102, Australia

**Keywords:** Aboriginal, Indigenous, cancer, cancer patients, radiotherapy, rural, regional, treatment

## Abstract

Travel logistics impede Aboriginal patients’ uptake of cancer treatments and is one reason for the poorer outcomes of Aboriginal people with cancer. This research examined benefits of a newly established rurally based radiotherapy unit in southwest Western Australia (WA), and included exploring the experience of Aboriginal patients and possible reasons for Aboriginal people’s underrepresentation in treatment. Semi-structured in-depth interviews with 21 service providers involved in the treatment and care of people with cancer, and 3 Aboriginal patients with cancer who undertook radiotherapy at the Service were undertaken. Data were subject to thematic analysis involving immersion in the data for familiarization, inductive coding, investigator discussion and refining of emerging themes and triangulation of patient and provider interviews. Aboriginal cancer patients were positive about the treatment and support they had received, highlighting the often complex challenges faced by rural Aboriginal cancer patients in accessing and maintaining treatment. Service providers offered suggestions for small numbers presenting to the Service, including late presentation, potential perceptions of cultural insensitivity on the part of service providers, out-of-pocket costs and under-ascertainment of Aboriginal status. The Service has put in place practices and initiatives to support patient health and wellbeing, including making the facility more welcoming towards Aboriginal people and ensuring culturally appropriate care.

## 1. Introduction

Despite progress in cancer diagnostic and treatment services in Australia, cancer patients living in regional and remote areas still have poorer outcomes than their urban counterparts [1,2,3,4]. Some of the factors leading to disparities in outcomes include more advanced cancer at diagnosis and greater challenges in access to and uptake of treatment [5]. In modern cancer treatment, radiotherapy is an important component of treatment options, shown to contribute to improving health outcomes and quality of care in people with cancer [5]. Due to the considerable infrastructure investment and the specialized care required, radiotherapy facilities tend to be located in metropolitan centers [6]. The significant logistical and financial challenges this presents for rural patients [5,7,8,9,10] can be a deterrent to them taking up radiotherapy treatment [11,12]. The resulting lower utilization of radiotherapy among rural cancer patients has been identified as a contributory factor to the poorer outcomes they experience [4].

Aboriginal and Torres Strait Islander (hereafter respectfully referred to as Aboriginal) cancer patients face specific barriers to accessing cancer care. These include fear or lack of trust of mainstream health facilities, lack of understanding or respect shown by health care providers and fatalistic or differing cultural beliefs about cancer [13,14]. These factors influence the extent to which Aboriginal people present for diagnosis and continue with treatment [15], contributing to the poorer outcomes and higher mortality experienced by Aboriginal cancer patients. [16,17,18,19] In addition, Aboriginal people living in rural areas are often reluctant to leave their home, family and country in order to access city-based cancer services [20]. Therefore, the lack of regional radiotherapy facilities likely has a disproportionate impact on the wellbeing of Aboriginal cancer patients in rural areas.

The vast size of the state of Western Australia (WA) presents particular logistical challenges to the rural population requiring cancer treatment [21]. To increase access to radiotherapy treatment for the residents of the South West region, the South West Radiation Oncology Service (the Service) was established in 2011 becoming the first regional radiotherapy service in WA [22]. Previously, all rural residents with cancer in WA had to travel to Perth to access radiotherapy treatment. Located in Bunbury, a regional city 179 km south of Perth with a population of approximately 33,075 people [23], the Service treats approximately 550 patients per year [22].

The Service is operated by a private provider of radiotherapy services, which works in partnership with two established hospitals—the regional public hospital and a private hospital - and independent medical specialists, all co-located on the Bunbury Health Campus. This local health network offers components of cancer services, such as allied health, medical oncology, surgical oncology, palliative care and investigational services, and operates in accordance with the public/private funding model that exists within Australia’s healthcare system. In addition to housing two radiotherapy linear accelerators, the Service contains consultation and nursing facilities, treatment planning and delivery facilities, with a radiation oncologist based locally. Approximately 70% of all cancer patients who need radiotherapy can be treated at the Service but more complex cases and pediatric radiotherapy continue to be referred to Perth for treatment [22].

In 2010, the Aboriginal population of the South West region was 3252, comprising 2% of the total population [24]. This is lower than national figures which indicate that approximately 3.1% of the entire Australian population identified as Aboriginal [25]. However, in the financial year 2014–2015, only 0.8% of radiotherapy treatment courses in Australia were for patients who identified as being Aboriginal [26]. One reason for this underrepresentation could be attributed to inaccurate recording as the Aboriginal status of the patient was not reported in a relatively high proportion of radiotherapy courses in the public sector (34%), with a particularly high ‘not stated’ rate (78%) in the private sector. The percentage for WA (44%) exceeded the national figure; figures from private providers in WA were unavailable [26].

This research explored the personal experiences of three Aboriginal cancer patients from the South West region, all of whom had under-gone radiotherapy treatment at the Service. We also examined observations made by health service providers about Aboriginal patients specific to their care at the Service. In so doing, our aim was to explore the perceptions of providers that Aboriginal patients were underrepresented at the Service and to explore any reasons and possible solutions to addressing this situation.

## 2. Materials and Methods

This primarily qualitative research utilized interviews with service providers and people with cancer and included assessment of some written material.

Ethics approval was granted by Human Research Ethics Committee at The University of Western Australia (RA/4/1/7384), the Western Australia Country Health Service (WACHS) Research Ethics Committee (2015:20) and the Western Australian Aboriginal Health Ethics Committee (483).

### 2.1. Sampling and Recruitment

Two types of participants were recruited: Aboriginal residents in the South West of WA who had been diagnosed with cancer since 2011 for whom radiotherapy was recommended, and service providers involved in treatment and care of people with cancer, including professionals from primary care, chemotherapy, radiotherapy, allied health, and cancer support services.

Particular efforts were made to recruit Aboriginal patients. Patients were invited to participate by the South West Aboriginal Medical Service (SWAMS). Investigators also asked staff at the Service and the local Cancer Council WA support service to identify and introduce some Aboriginal patients with cancer. As there were few Aboriginal patients initially identified, further attempts were made to identify additional participants.

Service providers were approached through personal invitation from colleagues or investigators. A meeting at the Service provided study information, and information was circulated by email and through flyers.

### 2.2. Data Collection

*Interviews:* Data was collected through semi-structured in-depth interviews, which were audio recorded with the participant’s permission. Interviews loosely followed an interview guide, one developed to explore relevant issues for cancer patients, and another for service providers. A participant information form and consent form were provided to all participants prior to the interview. Consent was recorded verbally in the case of telephone interviews with written consent obtained after the interview. Demographic information was collected via a short questionnaire at the conclusion of the interview.

Interviews were generally undertaken by two members of the research team at a venue suitable for the participant, facilitating debriefing, reflections and discussion following the interview. Patients were encouraged to bring a friend or relative to accompany them to the interview if they desired and were invited to ‘tell their story’ with a view to capturing their understanding of their overall treatment experience. For patients, the interviews explored their cancer treatment, including their experience of radiotherapy (and why they had opted to take up radiotherapy), why they had radiotherapy in Perth or Bunbury, their awareness/opinion of the Service, any barriers or facilitators to access, including the support they experienced and any opportunities for improvement. Service providers were asked for their views on a range of issues, specifically any issues they had encountered when caring for Aboriginal people with cancer including barriers and facilitators of care, and to provide any insights on the reasons for low numbers of Aboriginal cancer patients presenting.

*Data on patient numbers:* Patient Service related data was requested and obtained from the South West Radiation Oncology Service so that patient numbers over time could be examined, including the number of Aboriginal patients treated at the Service.

### 2.3. Data Analysis

Recorded interviews were transcribed verbatim and imported into the QSR International’s NVivo 10 qualitative data analysis Software to assist with data management. Data analysis followed the steps described by Green et al. [27] as follows: immersion in the data for familiarization, open coding, creating categories and identifying themes. Immersion required reading and rereading of the transcripts to understand what was said and to generate ideas about possible descriptive coding categories from the data. Service provider and patient interview transcripts were both initially coded by the one experienced coder and key concerns and issues identified. An inductive approach was used to identify recurrent categories, which were ordered, recorded and stored as ‘nodes’, with categories refined and grouped. The emergent thematic framework was discussed and agreed among the team involved in the interviews, including reading of the transcripts. Rigor was enhanced through a systematic approach, member checking, triangulation of service provider and patient interviews, coding validation and peer debriefing. Team members agreed on the key themes (and informative name for each theme) summarized under headings in the Results section.

## 3. Results

A total of 21 service providers were interviewed, 12 of whom were female and nine male. This group included five nurses, eight doctors (consisting of three specialists and five general practitioners (GPs)), four staff/volunteers from support organizations, an allied health professional, an Aboriginal Health Worker, a radiation therapist and a senior manager. In addition, service providers were drawn from the adjacent private hospital (St. John of God Bunbury Hospital), WACHS, SWAMS, Cancer Council WA, a provider of complementary therapies (SolarisCare) and a number of GP practices. Service provider interviews were carried out at their place of employment or, when more convenient, by telephone.

Three Aboriginal patients who were completing or had recently completed radiotherapy at the Service were interviewed. One patient was interviewed at Cancer Council WA’s patient support center in Bunbury with her mother in attendance for support. A second patient was interviewed in a health service provider’s office at the Service. His two sisters were present on the day of their brother’s interview and agreed to participate. This interview took place separately from the interview with their brother. A third patient was interviewed via telephone. One additional patient who agreed to participate was too unwell to be interviewed at the time. A brief summary of each of the three patients is set out below. Pseudonyms have been used to protect the identities of participants.

### 3.1. Patient Summaries

#### 3.1.1. Jim, 58 Years Old

Jim had been diagnosed with cancer seven months before the date of the interview and was coming to the end of an eight-week course of radiotherapy treatment. He admitted that he lacked understanding of radiotherapy. He found not being able to see the treatment, or feel any signs of improvement, difficult to come to terms with: *… you still can’t feel a thing and you wonder, ‘Is this doing any good?’*

Jim lived alone, in a smaller town about 60 km from Bunbury. He had some support in his home town, which included a GP, a local Aboriginal Health Service, a sister and many friends.

Jim was complimentary about staff at the Service and was comfortable talking to the staff about any concerns he might have regarding his treatment, with the time taken to talk with him and build the relationship clearly valued: *Well, I have a yarn with him and then I have a yarn with the doctor who operates that, the nurse, the young male nurses*. His main worries were about his ongoing needs when he returned home, particularly in relation to having enough food and the upkeep around his home. His two sisters were also concerned about the impact that Jim’s declining health may have on his ability to care for himself.

Jim was unable to drive, did not have access to daily transport and he was not eligible for travel support under Patient Assisted Travel Scheme (PATS) because he lived within 100 km of the treatment center. He was given information at the Service about a new privately-owned facility that was established to provide low cost (or free) accommodation for people needing to stay close to Bunbury for long-term treatments they were receiving there. Jim, who was accompanied by his sister, was able to stay at the accommodation for the duration of his treatment, which he described as “beautiful”. The local GP Down South and Aboriginal Health Service provided him with financial assistance to help pay for the accommodation. Staff from the local patient support services of Cancer Council WA assisted with transport to enable him get to and from his daily treatments when his sisters were unable to do so. 

#### 3.1.2. Justine, 25 Years Old

Justine had been diagnosed with a rare form of cancer, four months before the date of the interview. She initially had surgery and then a five-week course of radiotherapy, which had finished about a month before the date of the interview. She admitted to panicking the first time she had it (radiotherapy). In six weeks’ time she would find out the next steps in treatment, which might involve a further course of radiotherapy treatment.

Justine lived in Bunbury with her young daughter who was of primary school age. She had a supportive mother who helped to care for her daughter and accompanied her to medical appointments.

Justine was very happy with the treatment and care she had received at the Service. The availability of the radiotherapy service locally meant that she did not have to travel to Perth for this treatment and be away from her daughter. She reported that all the staff at the Service (from the receptionists to the medical staff) were friendly and helpful, scheduling appointment times to allow Justine to take her daughter to and from school.

Justine’s complicated cancer diagnosis required additional specialist medical care in Perth. Her mother drove her to Perth for these appointments and sometimes these trips required an overnight stay in a motel in Perth. She received financial assistance through the PATS scheme for petrol and accommodation. South West Aboriginal Medical Service assisted with the required paperwork and provided financial support for some of her medical bills. The Aboriginal Health Worker from SWAMS also provided her with socio-emotional support by accompanying her to a treatment session.

She found the staff at the local patient support center of Cancer Council WA to be particularly supportive and enjoyed attending physical activity classes there. They also responded to the needs of her daughter, arranging a tutor for the schooling she had missed because of her mother’s illness and with other acts of kindness: *She was behind in a bit of work at school. Like, because I was diagnosed with cancer I had to go back and forth to Perth and she got behind in a bit of work, and they helped with a bit of tutoring.* As well as appreciating the support at the health services, she appreciated the informal help and advice received through the Cancer Council WA’s service Dot’s Place: *I find Dot’s Place is really helpful. Yeah, they are really good here. They helped me, plus my daughter; yeah, they are really good. I like it.*

#### 3.1.3. Rosie, 58 Years Old

Rosie had a rare form of cancer, which had been diagnosed two and a half years before the date of the interview. She had had chemotherapy and had undergone a six-week course of radiotherapy. A few months after the initial diagnosis, she had ovarian tumors removed, which were found to be benign. She now had six-monthly check-ups.

Rosie lived alone in a small town approximately 30 km from Bunbury. She had five daughters who lived close by and appreciated the importance of having this family support. As she stated: *If you haven’t got family, then you are buggered.*

She stated that she was ‘lucky’ to have her treatment at the recently established local radiotherapy service in Bunbury, which meant that she did not have to go to Perth, which would have been associated with accommodation and financial worries: *A lot of people haven’t got family up there (Perth) you know, that can house them. You have to find accommodation and that costs money…and then you have got to get your treatment.*

She enthusiastically endorsed the service provided through the Service and praised the supportive staff: *The sisters there at radiation helped me… I can’t thank them enough*.

Rosie was provided with support from local services—her daily transport from her home to the Service was provided primarily by SWAMS and staff from the local patient support services of Cancer Council WA were also very welcoming and supportive. They would telephone her regularly to see how she was and invite her to come in for *a coffee or just a chat*. This support service also provided financial support towards her power bills, which she had really appreciated.

### 3.2. Number of Aboriginal Patients 

Based upon service data provided, the number of patients being treated at the Service each year since its opening, including those identifying as Aboriginal, is shown in Table 1. The figures show that Aboriginal people have consistently been underrepresented in attendances at the Service based on their numbers in the overall population of the South West.

### 3.3. Reasons for Low Numbers

The underrepresentation of Aboriginal people among cancer patients treated at the service, as shown in the above statistics, was confirmed by service providers who reported seeing very few Aboriginal patients. They were uncertain why this was the case, but offered some suggestions, which are summarized below.

#### 3.3.1. Late Presentation to General Practitioner

Service providers suggested that some Aboriginal people may be reluctant to present to their GP when cancer symptoms first occur. By the time that they do attend for consultation, their cancer is so far advanced that radiotherapy treatment is no longer an option.

Reasons suggested for delays in seeking medical advice include traditional Aboriginal beliefs around cancer and cancer treatment. The beliefs identified by our participants as being commonly held by Aboriginal people were that a cancer diagnosis is synonymous with a death sentence. A further reason identified for late presentation was that Aboriginal people may experience many life stressors, including multiple health care issues. As a result, cancer symptoms and the prospect of diagnosis can be overwhelming and difficult to priorities for attention. A third reason identified for not seeking timely medical assistance was a desire for privacy and reluctance to reveal their illness to others.

#### 3.3.2. Cultural Insensitivity

A health service provider, keen to address the issue of low numbers, questioned whether cultural sensitivity of health service staff was a factor: *… it would be tragic to find out that the Aboriginal patients were being referred but weren’t attending because they didn’t feel culturally safe* (male_age not provided).

One health service provider gave an example of an Aboriginal patient whose treatment plan included both chemotherapy and radiotherapy. As an inpatient having chemotherapy treatment the patient felt badly treated in both manner and voice by a member of the health staff and, as a result, initially refused any further form of treatment. Following the intervention of an Aboriginal health worker who involved the radiation oncologist to explore other treatment options with the patient, the treatment was subsequently recommenced.

#### 3.3.3. Out-of-Pocket Costs

Navigating the public/private system between the private hospital and the Service was raised as a possible reason why some Aboriginal patients may not present for treatment at the Service. Some patients preferred to go to Perth because all treatment services (in relation to certain scans and tests) were unambiguously covered within the public health system there, which was not necessarily the case in Bunbury. As one health service provider stated: *… because some things are public and some things are private it is confusing to navigate, and so in sort of trying to navigate they just say, ‘Let’s go to one place where everything exists and it is all in one hospital and all public’* (male_36 yrs).

#### 3.3.4. Non-Reporting of Aboriginal Status 

In addition to the above points for why Aboriginal patients are not seen in any numbers at the Service, it was proposed that perhaps Aboriginal patients are attending but that these attendances are not reflected in the statistics. This may be because patients are not identifying as Aboriginal or because their status is not being recorded due to inadequate processes. A staff member with responsibility for data monitoring suggested that there is under reporting of Aboriginal status. 

### 3.4. Measures to Address Low Numbers

A number of measures had been put in place as part of efforts to increase the number of Aboriginal patients presenting for treatment at the Service. These included raising awareness of the Service among Aboriginal patients, ensuring that they are made to feel welcome and, when they do attend, that their Aboriginal status is captured.

#### 3.4.1. Community Awareness Activities

Service providers reported that the Service is working hard to ensure it is fully engaged with the Aboriginal community in the South West region. Information-raising events, such as talks and presentations, are provided to Aboriginal community groups to increase awareness of the Service and improve perceptions around its accessibility.

There were also activities held to raise awareness on cancer prevention and management. These are intended to make Aboriginal people more aware of the signs and symptoms of cancer and the importance of early diagnosis to increase the effectiveness of treatments (including radiotherapy).

Presence at key community events and education with community groups is considered essential to building relationships and breaking down barriers. Education sessions at Men’s Groups, around general health measures and what is radiotherapy, have been carried out. With the help of the McGrath Breast Cancer Nurses, talks on breast cancer health and screening have been provided to the Busselton Women’s Group. These are tailored to be culturally appropriate with female leaders carrying out the education sessions. Community events that are aimed at the whole community, such as Seniors Day, have been attended by oncology health care providers (such as the Prostate Cancer Foundation, Cancer Council WA) so that there is a coordinated presence at these events.

#### 3.4.2. Culturally Appropriate Care

Service providers noted the teamwork in place between the various disciplines, both within the campus and externally, with team members collaborating to treat and support Aboriginal patients. Multidisciplinary team meetings are inclusive with Aboriginal health staff from both the public and the private hospitals and SWAMS invited to attend. The good working relationship between the Aboriginal health worker with responsibility for cancer patients at SWAMS and the head radiation oncologist was highlighted with both parties working collaboratively with the patient to develop the best treatment program. When there was co-location of SWAMS on the South West Health Campus close to where the Service is located, a good working relationship had been established that had benefits for both cancer education and patient support.

The time spent by staff based at the health campus in developing a good rapport with their patients was particularly noted. As Justine said: *I found the people there (the Service) were really supportive. Every morning or every day I would go in they would be, ‘Hello! How are you? How’s your day been?’ I would have a chat with them before I go in*. An Aboriginal health worker told of a patient who had felt that he was not receiving appropriate treatment because he was a ‘black bastard’. A specialist cancer nurse took time to sit with the patient, explain the situation and outline further treatment options. The Aboriginal health worker subsequently expressed appreciation to the nurse on behalf of the patient, demonstrating the importance to the patient of having culturally aware staff and the role of the Aboriginal health worker acting as a conduit between the nurse and patient.

The Service had committed to cultural awareness training for all staff members to ensure that they act in a culturally appropriate manner when meeting Aboriginal clients and their families.

#### 3.4.3. Improved Recording of Status

Staff at the Service reported efforts being made to improve recording of Aboriginal status. At first attendance, a patient is asked to complete a patient registration form where there is a section specifically regarding Aboriginal status. If the person identifies as Aboriginal, nursing staff offer a handout from the National Centre for Aboriginal and Torres Strait Islander Statistics. There are information brochures available at reception explaining the rationale for the question on Aboriginal identity and a poster from the Australian Bureau of Statistics highlighting the importance of establishing Aboriginal status is also displayed.

## 4. Discussion

Regional radiotherapy centers benefit rural patients in terms of improved use of radiotherapy treatment [6,12], however, the numbers of Aboriginal patients treated at a newly established service in the South West region of WA have been low. This research set out to identify reasons for this underrepresentation and offer possible solutions to address this situation. We made considerable efforts to recruit Aboriginal patients who had been offered radiotherapy, but only small numbers were identified with one other potential patient too unwell when contacted and therefore unavailable for interview. Despite interviewing only a small number of Aboriginal participants, those Aboriginal cancer patients interviewed who were treated at the Service were positive about the treatment and additional support they had received. Service providers were unsure why small numbers presented, but offered some suggestions including late presentation to a GP, perceptions of cultural insensitivity on the part of service providers and out-of-pocket costs incurred. The mixed cost of cancer care in Bunbury, with regard to investigations and treatment perspective, is probably influential on patients attending the service as Aboriginal health care providers and patients would understandably try to minimize the financial burden on themselves. It was also thought that Aboriginal patients may in fact be attending the Service but their Aboriginal status is not being captured.

To encourage attendance by Aboriginal patients, the Service has put in place practices and initiatives that aim to make the facility more welcoming towards Aboriginal people and allay any fears that patients may have about receiving radiotherapy treatment. These include community-based awareness raising events, which promote the Service and the benefits of radiotherapy for cancer treatment. Efforts are also made to ensure culturally appropriate care is provided at the Service at all times. These practices and initiatives appear to be working to an extent, based on recent numbers of Aboriginal patients. However, further work is required. Fear and lack of understanding of radiotherapy treatment have been found to feature prominently in consumers’ decisions regarding the utilization of radiotherapy [11]. There is limited evidence that is specific to Aboriginal patients but our findings confirm these suggestions and underline the need for information resources, support services and interventions to increase awareness of radiotherapy. One study which examined Aboriginal participation in radiotherapy treatment showed compliance levels on a par with non-Aboriginal patients [28]. This suggests that when Aboriginal patients do engage in radiotherapy, their levels of satisfaction with the treatment are comparable with those of non-Aboriginal patients and that it is the pre-treatment phase of care that is the most difficult and presents most challenges.

Cancer patients often experience many stressors in addition to their cancer diagnosis, however financial worries, dependent family members and other health issues were all concerns identified by the Aboriginal participants in our study which may disproportionately impact Aboriginal people. While the provision of a regional radiotherapy service addresses some practical barriers for Aboriginal people to access cancer care, [5,20] other ‘life priorities’ [29] may take precedence and impede attendance at medical appointments. Furthermore, certain beliefs that are widely held in Aboriginal culture may affect decisions around accessing cancer services. These beliefs are consistent with low levels of health literacy in relation to cancer generally among Aboriginal people [30]. It is vital that Aboriginal people are adequately educated to understand the importance of identifying cancer symptoms and supported to seek timely medical advice because radiotherapy (as with other treatments) has been found to have the best curative effect in the early stages of cancer [5]. It is important to note here that the failure of providers to recognize, sometimes as a result of other comorbidities, that symptoms which Aboriginal people present to them with are cancer-related, may also result in a delay in diagnosis [31]. Sadly, since many Aboriginal people present late in the course of their illness there is less time available to seek their input into health services research.

A further reason why Aboriginal patients are not presenting to the healthcare system may be because of culturally insensitive behavior demonstrated by health professionals in previous encounters. This was suggested by some service providers in our study but no incidences of culturally insensitive behavior were reported by patients. This positive outcome may reflect increasing awareness and education of health professionals around cultural sensitivity. In addition, it may be attributed to leadership within the Service, which emphasizes the importance of patient centered care and respect for Aboriginal people and patients. These are key enablers for health professionals to communicate effectively and provide quality care [32,33,34].

All the patients interviewed noted the support they had received from local Aboriginal health workers and Aboriginal-specific support services. Aboriginal health workers, who have both community and clinical knowledge, provide culturally safe health care to Aboriginal patients by advocating for patients and educating other health professionals on the delivery of culturally safe care [15,35,36,37,38]. The value added by Aboriginal employees is most evident in the role they play in helping to negotiate the disparate social and knowledge systems involved when traditional and western health systems interface [38]. However, it is important that the delivery of culturally appropriate treatment and support is not seen as just the responsibility of Aboriginal staff. All health care providers must understand their shared responsibility in making Aboriginal patients and families feel culturally safe and welcome [39]. Cultural appropriateness of an entire health service has been identified as a key factor influencing the extent to which Aboriginal Australians present for diagnosis and continue with treatment [15]. 

The issue of costs resulting from treatment services was not raised by the patients described here, but the implications of costs associated with support and the importance of meeting support needs were evident. Our study found that although all our participants had received considerable support with travel from family (as was the case with all their support needs) the additional assistance that was offered by support organizations proved to be invaluable. Assistance with accommodation and other financial support had also alleviated some of the burden of treatment for participants. The socio-economic disadvantage and poorer health literacy of many Aboriginal people [40] necessitates greater levels of support, so it is particularly important that support systems facilitate their care through treatment to discharge and beyond. A gap in the ongoing support structures was identified by Jim, who expressed concern about how his post-treatment needs would be met. The challenges faced by rural cancer survivors accessing follow-up care is a widespread concern [41,42,43] but the unmet needs of Aboriginal survivors are particularly significant [44]. Enhanced discharge planning arrangements need to be established for follow-up support to address the ongoing needs of rural Aboriginal cancer patients.

A further area requiring attention, acknowledged by service providers, related to the recording of Aboriginal status. Weaknesses in current processes for capturing this information could mean that Aboriginal patients are attending the Service in greater numbers than the figures suggest. Under-recording of Aboriginal patients is a wide scale issue. For example, in 2011–2012 the estimated ‘true’ number of admissions for Aboriginal persons to Australian public hospitals was found to be approximately 12% higher than reported [45]. One reason for this discrepancy is that patients may choose not to identify, possibly fearing that to do so may influence service providers’ perceptions. These concerns may be unfounded or may be the result of previous negative experiences with health professionals. Whatever the reason, it reinforces the need for staff to be culturally competent [32,33,34] and for training of any staff involved in the collection of patient information in the appropriate collection and recording of Aboriginal status. Without high quality data, the inequalities in cancer outcomes for Aboriginal Australians cannot be understood [46].

## 5. Conclusions

Though all patients in our study had different diagnoses and needs, there were a number of common factors that emerged in their cancer experiences. Their stories serve to highlight the often complex challenges faced by rural Aboriginal cancer patients in accessing and maintaining treatment. While many of the challenges faced by Aboriginal patients in rural areas are similar to those of other rural residents, the socio-economic disadvantage and poorer health literacy experienced by many Aboriginal people impose an additional overlay impeding access to radiotherapy treatment. The low number of Aboriginal patients presenting at the Service is a concern and suggests that the Aboriginal community is not benefitting from the regional treatment facility to the same extent as the wider community. However, when Aboriginal patients do engage in radiotherapy, their levels of satisfaction with the treatment are comparable with those of non-Aboriginal patients.

To engage Aboriginal people in radiotherapy treatment, a service must offer a supportive environment that addresses the access challenges faced by rural Aboriginal cancer patients from initial diagnosis through to follow-up care. The positive reports from participants in this study indicate that the Service has, to a large extent, succeeded in creating such an environment, with service providers both within the campus and externally collaborating to treat and support Aboriginal patients. It is hoped that these experiences, together with the ongoing promotional efforts of the Service, will lead to greater numbers of Aboriginal people from the South West region making use of the radiotherapy services that are available locally.

Our findings are based on a limited sample of Aboriginal patients and further research to confirm and expand on this work is needed given the limited number of studies investigating the attitudes of Aboriginal cancer patients towards radiotherapy and uptake of the treatment.

## Figures and Tables

**Table 1:** Percentage of Southwest radiotherapy patients identifying as Aboriginal, by year.

Year	Number of	Number of	% Of All Patients
	Aboriginal Patients	Non-Aboriginal Patients	
2011–2012	1	292	0.3
2012–2013	2	451	0.4
2013–2014	0	507	0.0
2014–2015	1	512	0.2
2015–2016	4	507	0.8
2016–2017	2	660	0.3
